# Next-Generation Immunohistochemistry in Thyroid Neoplasm: A Practical Review on the Applications in Diagnosis and Molecular Classification

**DOI:** 10.1007/s12022-025-09851-6

**Published:** 2025-03-20

**Authors:** Jonathan P. Rivera, Jen-Fan Hang

**Affiliations:** 1 Department of Laboratories, Philippine General Hospital, Manila, Philippines; 2 Department of Pathology and Laboratory Medicine, Taipei Veterans General Hospital, Shipai Rd, No. 201, Sec. 2, Taipei, 11217 Taiwan; 3 Department of Pathology, School of Medicine, National Yang Ming Chiao Tung University, Taipei, Taiwan; 4 Institute of Clinical Medicine, National Yang Ming Chiao Tung University, Taipei, Taiwan

**Keywords:** Immunohistochemistry, Thyroid, BRAF VE1, RAS Q61R, Pan-TRK, ALK, PTEN, β-Catenin

## Abstract

An integrative histologic and molecular classification of thyroid tumors has become clinically relevant due to the potential role in risk stratification and selection of targeted therapy. In this review, we discuss the applications of six “next-generation” immunohistochemical markers, namely BRAF V600E (clone VE1), RAS Q61R (clone SP174), pan-TRK (clone EPR 17341), ALK (clones 5A4 or D5F3), PTEN, and β-catenin in the pathologic diagnosis and molecular classification of thyroid tumors. These biomarkers allow the in situ examination of tumor tissue and assist in the diagnosis and pathologic staging by highlighting tumor border and patterns of invasion, identifying isolated tumor cells in lymph nodes, distinguishing lymph node metastasis from benign intranodal thyroid inclusions, and diagnosing multicentric thyroid carcinomas with discordant molecular drivers. Furthermore, it can identify specific thyroid neoplasms that may occur sporadically or may be associated with hereditary syndromes. The next-generation immunohistochemistry provides a novel solution to challenging issues in thyroid pathology and fast turn-around time for accurate molecular classification and further guidance of therapeutic management.

## Introduction

Thyroid cancer is the most common endocrine malignancy worldwide. The Cancer Genome Atlas (TCGA) project molecularly classified thyroid carcinomas into a spectrum of *BRAF*-like and *RAS*-like tumors, with the rare kinase gene fusion-associated carcinomas largely positioned midway or closer to the *BRAF*-like tumors [1]. In addition, the molecular drivers of thyroid carcinoma were elucidated and found to be mutually exclusive, expressed in all tumor cells, and correlate well with morphology and biological behavior. These key gene alterations have become prime targets of the emerging selective kinase inhibitors with promising therapeutic results, redefining the treatment of advanced and iodine-refractory thyroid cancers [2, 3]. Likewise, predisposing germline mutations in key tumor suppressor genes are associated with familial hereditary syndromes, which can drive the development of thyroid lesions and neoplasms [4]. Thus, an integrative molecular and histopathologic approach to classifying thyroid tumors is becoming necessary in clinical practice.

Immunohistochemistry (IHC) plays an essential role in pathologic diagnosis, from identifying lineage-specific protein expression to demonstrating surrogate biomarkers of tumor-defining gene alterations. The newer assays, dubbed “next-generation IHC,” have allowed for a more precise classification of tumors, identification of potential therapeutic targets, and the screening of patients for underlying hereditary syndromes with lower costs and faster laboratory turn-around time than most molecular assays [5]. Another advantage is that IHC is not compromised by the suboptimal quality of nucleic acid in formalin-fixed paraffin-embedded tumor tissues, an issue commonly encountered in molecular assays. Furthermore, IHC allows the in situ examination of tumor cells, enabling pathologists to directly correlate morphology with the immunohistochemical expression.

In this review, we discuss the applications of six “next-generation” immunohistochemical markers for thyroid pathology. Mutation-specific antibodies using BRAF V600E (clone VE1) and RAS Q61R (clone SP174) are suitable for identifying the most prevalent mutations in thyroid tumors, while pan-TRK (clone EPR 17341) and ALK (cloned 5A4 and D5F3) are used to detect the overexpression of chimeric protein products resulting from kinase gene fusions, present in a small but clinically relevant subset of thyroid carcinomas. Similarly, PTEN and β-catenin IHC are suitable surrogate markers for the underlying gene alterations in specific thyroid neoplasms, which may occur sporadically or be associated with corresponding hereditary syndromes. We aim to review the performance of these immunohistochemical stains in the molecular classification of thyroid carcinomas, highlight their specific applications that we find extremely useful in diagnosing or staging tumors, and demonstrate the important clinical scenarios that pathologists should be aware of when incorporating them into their practice.

## Next-Generation IHC with Applications in Diagnosis and Targeted Therapy

### BRAF V600E (Clone VE1)

**Fig. 1:**
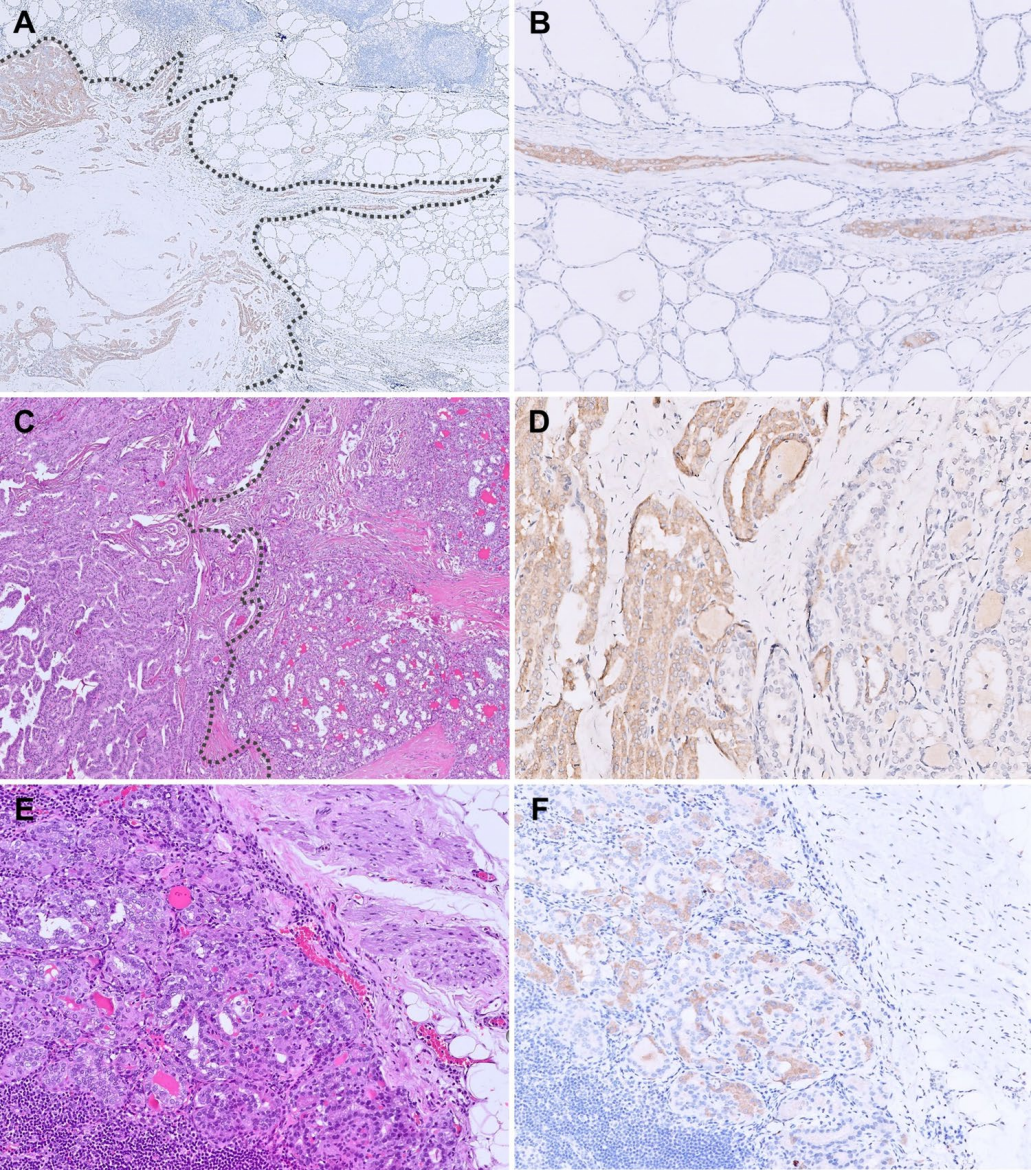
BRAF V600E IHC in diagnosing PTCs. **A** BRAF IHC highlights the tumor extent of this classic PTC (20 ×). **B** It also highlights lateral tubular growth and isolated tumor clusters, which are unique patterns of invasion most frequently encountered in *BRAF* p.V600E-mutated PTCs (100 ×). **C** Collision of a *BRAF* p.V600E-mutated PTC (left of the dotted line) and a *RET::CCDC6* fusion-associated PTC (H&E, 50 ×). **D** BRAF IHC highlights the interface of the molecularly discordant tumors with variable intermingling of the neoplastic follicles in the center (200 ×). **E** Admixed lymph node metastasis consisting of molecularly discordant neoplastic microfollicles of *BRAF* p.V600E and *RET::CCDC6* fusion-associated PTCs (H&E, 100 ×). **F** BRAF IHC highlights the BRAF-positive neoplastic follicles, confirming the admixture of the molecularly discordant tumors in metastasis (100 ×)

*BRAF* p.V600E mutation is the most common molecular driver identified in papillary thyroid carcinomas (PTCs, 58.5–86.8%) [1, 6]. The mutated protein can be detected in the tumor cytoplasm using a mutation-specific antibody clone VE1, with excellent specificity (99–100%) and sensitivity (84–100%) across various tumor types, including thyroid cancers [7–10]. In our experience, BRAF V600E IHC is highly useful in highlighting the tumor extent and the unique patterns of invasion (lateral tubular growth and isolated tumor clusters), which are characteristic of *BRAF*-mutated PTCs (Fig. 1A and B) [11–13]. Identification of these patterns of invasion may have potential clinical significance as prior investigators have found them to be predictive of lymph node metastasis [11]. Furthermore, in well-encapsulated or well-defined thyroid tumors with PTC-like nuclear changes, BRAF V600E IHC can serve as an efficient diagnostic tool, as the presence of a *BRAF* p.V600E mutation is a desirable exclusionary criterion for the diagnosis of non-invasive follicular thyroid neoplasm with papillary-like nuclear features (NIFTP) [14].

Previous research has attempted to distinguish intraglandular tumor spreading of PTC from multicentric tumors by applying histologic criteria based on tumor size and morphology [12]. In our recent study, BRAF V600E IHC was utilized to identify multifocal PTCs with discordant molecular drivers (*BRAF*-positive and *BRAF*-negative tumors), supporting diagnoses of multicentric disease in these cases [13]. However, in multifocal PTCs where all foci harbor mutual *BRAF* p.V600E mutation, additional clonality studies are warranted to clarify whether these represent truly multicentric tumors or intraglandular tumor spreading.

The occurrence of subclonal BRAF expression and intratumoral heterogeneity in PTCs remains controversial. While some studies using molecular assays suggest that these phenomena are not uncommon [15–19], others, including TCGA, report them as exceedingly rare, if not nonexistent [1, 20, 21]. Additionally, rare cases of PTCs with concurrent multiple molecular drivers were thought to represent subclonal BRAF expression and intratumoral heterogeneity [1, 15, 16, 18, 21]. However, these studies were limited by methodologies that could not clarify whether these mutations occurred within the same tumor cells or in distinct tumor populations. In our previous studies, BRAF V600E IHC consistently demonstrated homogeneous cytoplasmic staining in *BRAF* p.V600E-mutant PTCs, except in rare cases of molecularly discordant PTCs in collision [6, 13]. In the latter cases, the advantage of next-generation IHC over traditional molecular assays is highlighted, as BRAF V600E IHC enabled the precise identification and demarcation of molecularly discordant PTCs (Fig. 1C and D) and distinguished their corresponding lymph node metastasis (Fig. 1E and F) [13]. This approach offers a novel insight into rare PTCs reported to have concurrent multiple molecular drivers, which are theoretically mutually exclusive. Likewise, it suggests that a subset of cases previously considered to represent *BRAF* subclonality or intratumoral heterogeneity may now be attributed to the possible collision of molecularly discordant PTCs [1, 13, 15, 16, 18, 21]. Ultimately, further studies are needed to better define and validate the methodologies for assessing *BRAF* subclonality. Nevertheless, rare cases of heterogeneous BRAF IHC expression may complicate histologic interpretation and influence treatment decisions. In such cases, meticulous correlation of staining patterns with morphologic features, adequate tumor sampling, review of the IHC protocol, and molecular confirmation are recommended. The accurate diagnosis of multifocal PTCs with discordant molecular drivers, including cases of molecularly distinct tumor collisions, is clinically relevant for selecting appropriate targeted kinase inhibitors, especially in advanced or radioiodine refractory disease, which occurs in 10–15% of PTCs [2, 22].

**Fig. 2:**
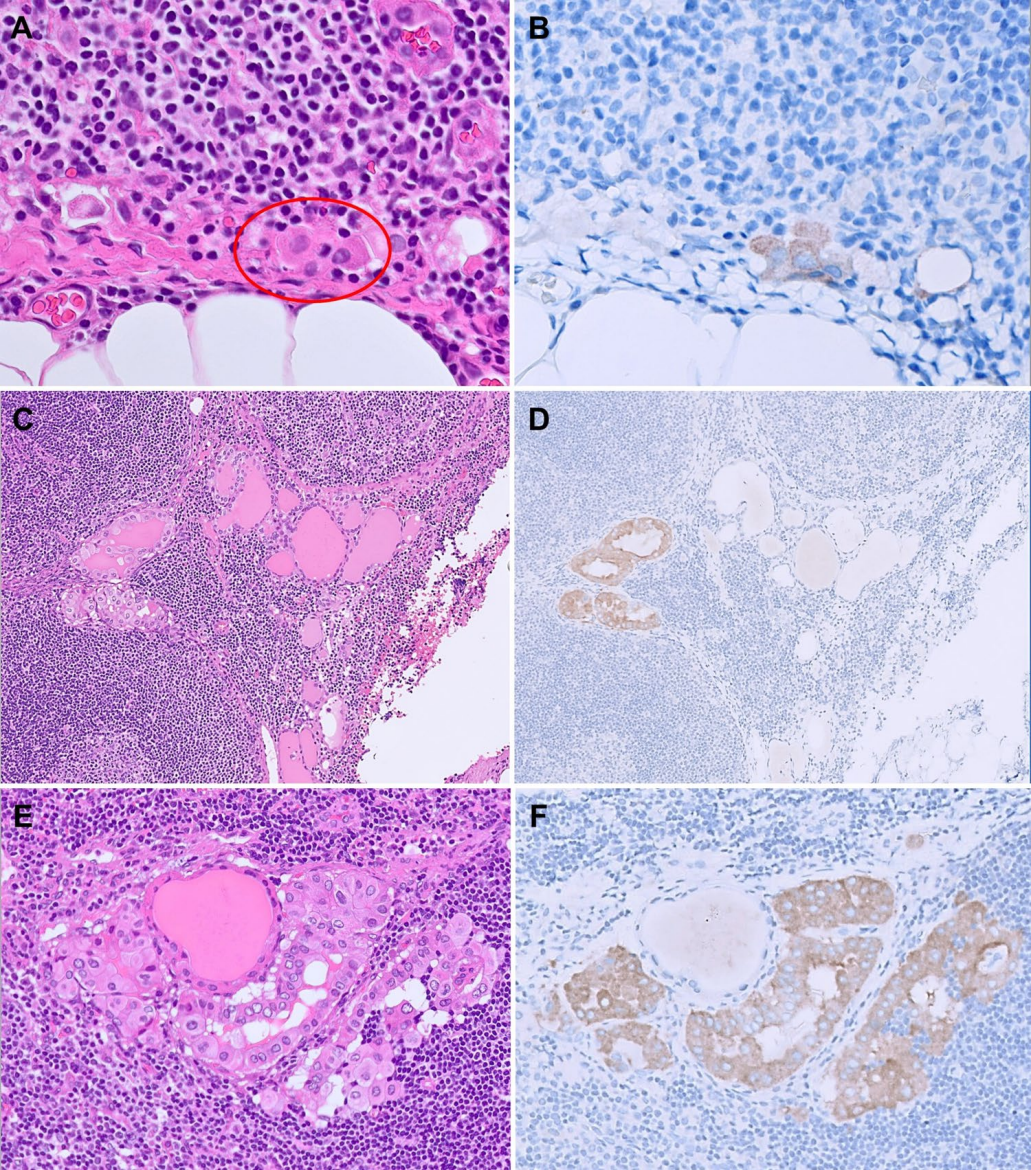
BRAF V600E IHC in assessing tumor deposits in lymph nodes. **A** Rare isolated tumor cells resembling histiocytes are present in the lymph node (H&E, 400 ×). **B** BRAF IHC highlights the isolated tumor cells (400 ×). **C** Thyroid follicles present in the lymph node of a patient with a primary *BRAF* p.V600E-mutant PTC (H&E, 100 ×). **D** BRAF IHC distinguishes the tumor deposits from nearby benign intranodal inclusions (100 ×). **E** Thyroid follicles with varying degrees of atypia are present in the lymph node of a patient with a primary *BRAF* p.V600E-mutant PTC (H&E, 200 ×). **F** BRAF IHC confirms that the deposit is an admixture of both metastatic PTC and benign intranodal thyroid inclusion (200 ×)

In the lymph nodes, BRAF V600E IHC can highlight metastatic isolated tumor cells or clusters, which could be missed on routine H&E evaluation (Fig. 2A and B). This has implications for accurate pathologic staging. In addition, the distinction between benign intranodal thyroid inclusions and metastatic thyroid carcinoma is controversial and poses a diagnostic challenge among pathologists. Traditionally, microscopic size, benign cytologic features, and absence of papillae, stromal desmoplasia, and psammoma bodies were regarded as morphologic features in favor of benign thyroid inclusions [23, 24]. However, in the absence of clinical and imaging findings of the thyroid, these morphologic features remain subjective and do not provide conclusive evidence for the non-neoplastic nature of the benign intrathyroidal follicles. BRAF V600E IHC serves as an objective tool to distinguish between benign intranodal inclusions and lymph node involvement of a *BRAF*-positive PTC. In our recent study applying BRAF V600E IHC, we found benign thyroid inclusions and metastatic deposits of PTC may co-exist in the same lymph node as separate aggregates (Fig. 2C and D) or as intimately admixed deposits (Fig. 2E and F), suggesting a potential biological link between these two [25]. This led us to further refine the diagnostic criteria for benign intranodal thyroid inclusions with the recommendation of BRAF V600E IHC for optimal sensitivity and specificity.

Lastly, it should be emphasized that the utility of BRAF VE1 IHC is limited to detecting only *BRAF* p.V600E-mutant PTCs. It does not identify PTCs with non-p.V600E *BRAF* mutations, including other class I mutations (p.V600K/M/R/D), class II and III mutations, or *BRAF* fusions [26].

### RAS Q61R (Clone SP174)

Mutations in the *RAS* family of oncogenes (*NRAS*, *HRAS*, and *KRAS*) are key molecular events in follicular-patterned neoplasms including follicular adenoma (FA, 20–25%), follicular thyroid carcinoma (FTC, 30–45%), and follicular variant of PTC (FVPTC, 30–45%), as well as high-grade tumors such as poorly differentiated thyroid carcinoma (20–40%) and anaplastic thyroid carcinoma (ATC, 20–30%) [27]. Similarly, NIFTP is found to be enriched with similar *RAS* mutations [28]. Furthermore, *RAS* mutations have also been found in thyroid nodules that would otherwise have been classified as hyperplastic based on morphology. From a diagnostic standpoint, these nodules could be more appropriately classified as FAs, given the presence of a driver mutation. However, in the absence of molecular information, distinguishing FA from these “hyperplastic-appearing” nodules remains largely subjective, especially in the setting of multinodular goiter. Importantly, this distinction has no clinical relevance following complete surgical resection [29]. Reflecting this evolving understanding, the latest WHO Classification of Endocrine and Neuroendocrine Tumors now favors the term thyroid follicular nodular disease over nodular hyperplasia or adenomatous hyperplasia [14]. This shift acknowledges that clonal nodules with oncogenic mutations can arise within multinodular goiter and may represent true neoplasms. Among the different *RAS* genes, mutations in *NRAS* are the most predominant (65–66%), followed by mutations in *HRAS* (21–27%) and *KRAS* (8–13%) [1, 30, 31]. The most frequent mutation is the substitution of glutamine by arginine in codon 61 (p.Q61R) of the *RAS* genes, representing 79–85%, 79–88%, and 25–60% of the *NRAS*, *HRAS*, and *KRAS* mutations, respectively [1, 31].

The pathologic classification of follicular-patterned tumors remains problematic as significant interobserver variability exists regarding the morphologic distinction of NIFTP and invasive encapsulated FVPTC from FA and FTC, and whether this pursuit still has clinical relevance [32]. More importantly, the dichotomization of PTCs by the TCGA project into *RAS*-like and *BRAF*-like PTCs confirms that most invasive encapsulated FVPTC and NIFTP are within the same family of *RAS*-like tumors and should be distinguished from the *BRAF*-like PTCs because of different tumor morphology, biological behavior, and response to radioiodine therapy [1]. This implies that in morphologically ambiguous follicular-patterned neoplasms, identifying the *RAS* mutation using next-generation IHC, in lieu of molecular analysis, offers a powerful tool in classifying these tumors into more clinically relevant groups and help obviate the existing subjectivity inherent in its morphological interpretation. Furthermore, once a *RAS*-like follicular-patterned neoplasm is identified, attention to its architecture becomes paramount to assess capsular and/or angioinvasion, which the IHC can help highlight, further improving diagnostic accuracy [1, 29].

**Fig. 3:**
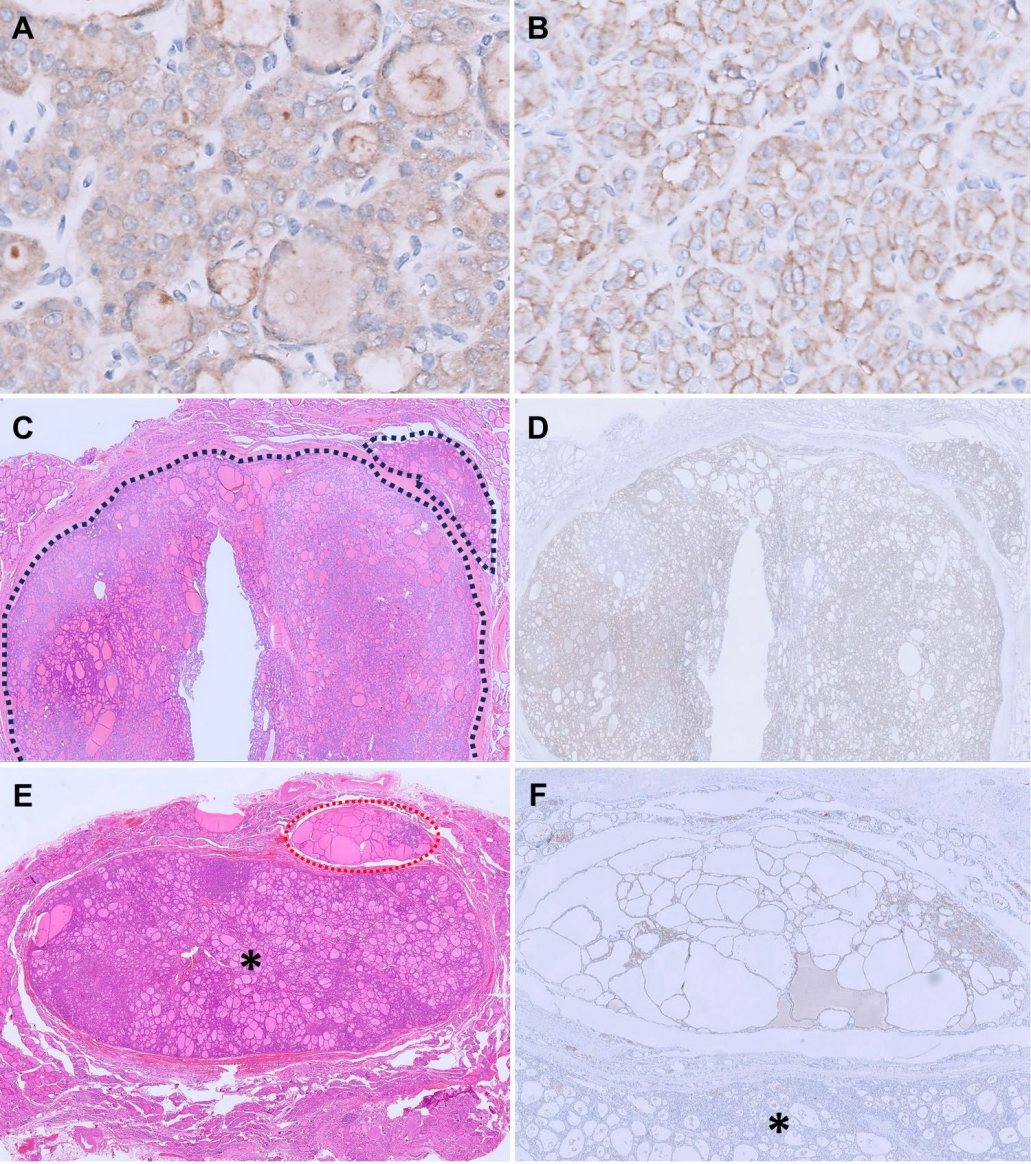
Utility of RAS Q61R IHC in diagnosing follicular neoplasms. **A** A *RAS*-mutant FA showing diffuse moderate cytoplasmic staining with RAS IHC (400 ×). **B** A *RAS*-mutant widely invasive FTC with a distinctive membranous staining pattern with RAS IHC (400 ×). **C** An encapsulated follicular neoplasm with a suspected invasive satellite nodule (H&E, 10 ×). **D** RAS IHC highlights the tumor and its satellite nodule, confirming the diagnosis of minimally invasive FTC (10 ×). **E** A nodule with morphologic features of follicular adenoma (asterisk) immediately adjacent to a “hyperplastic-appearing” nodule (H&E, 50 ×). **F** RAS IHC is positive in the smaller “hyperplastic-appearing” nodule and negative in the follicular adenoma, confirming that both are molecularly unrelated and excluding the possibility of an FTC with an invasive satellite nodule (200 ×)

**Fig. 4:**
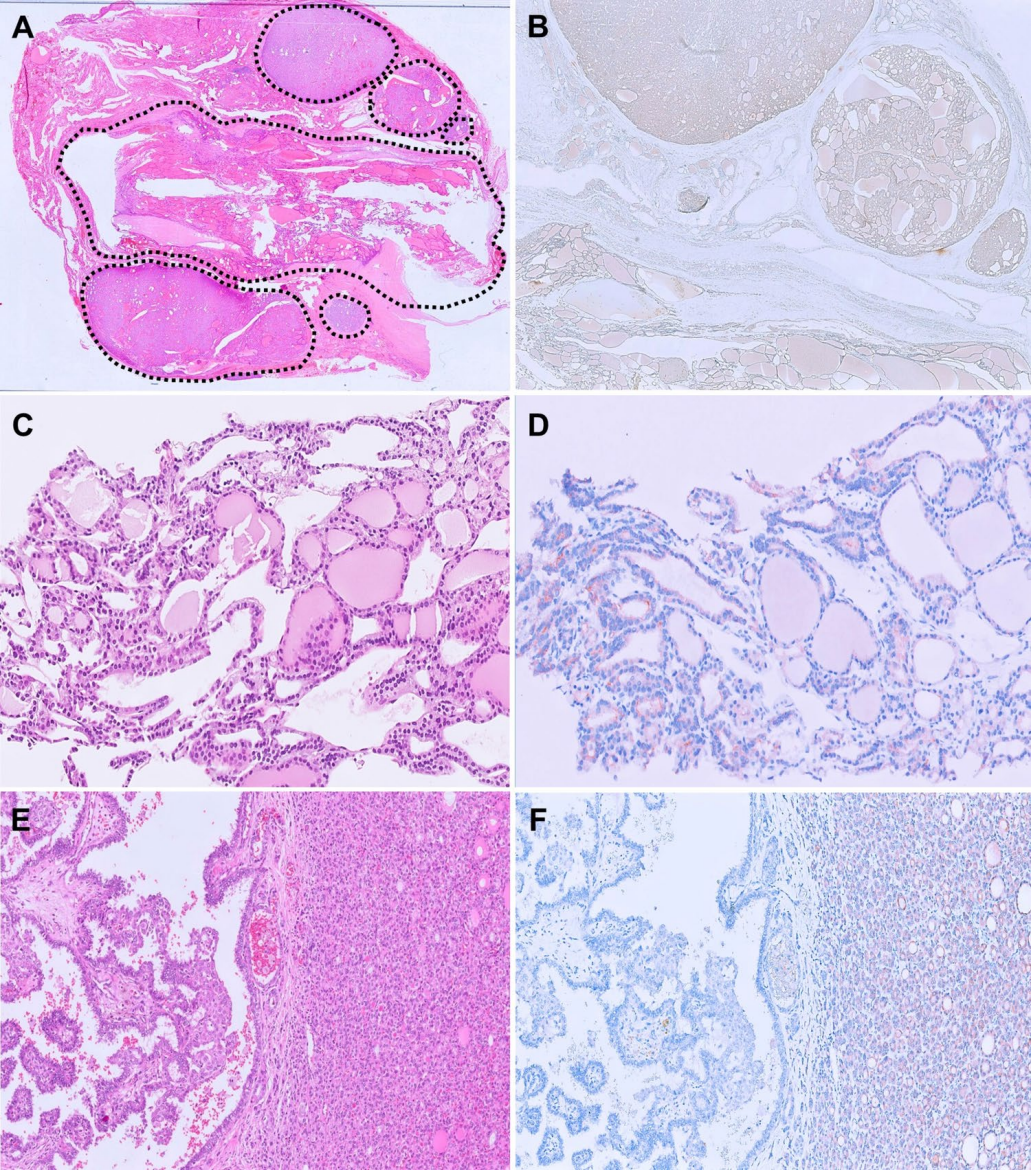
Utility of RAS Q61R IHC in diagnosing follicular neoplasms. **A** Widely invasive follicular thyroid carcinoma with a multinodular goiter-like appearance (H&E, 2.5 ×). **B** RAS IHC highlights the infiltrative nodules confirming the diagnosis (10 ×). **C** Cytoarchitecturally bland goiter-like metastasis of an FTC in a needle biopsy of the lung (H&E, 200 ×). **D** Staining with RAS IHC confirms the neoplastic nature of this metastatic lesion (200 ×). **E** Collision of a *RET::NCOA4* PTC on the left with a *RAS*-mutant follicular neoplasm on the right (H&E, 100 ×). **F** RAS IHC highlights the interface of the molecularly discordant tumors (100 ×)

The commercially available antibody SP174 effectively detects the RAS p.Q61R protein, demonstrating homogeneous moderate to strong granular cytoplasmic and/or membranous staining in tumor cells (Fig. 3A and B). It demonstrates high sensitivity (90.6%) and specificity (92.3%) for identifying Q61R mutations across all three *RAS* genes [29, 33–35]. In cases where non-specific cytoplasmic staining occurs in the background thyroid parenchyma, the presence of a membranous staining pattern can serve as a potential distinguishing feature to confirm that the tumor harbors clonal *RAS* p.Q61R mutation. In diagnosing follicular-patterned carcinomas, the IHC is valuable in delineating tumor border, infiltration, capsular and/or vascular invasion, and multifocality [33, 34]. In our experience, RAS Q61R IHC has been indispensable for confirming the diagnosis of minimally invasive FTC (Fig. 3C and D). Occasionally, follicular adenomas and hyperplastic nodules within follicular nodular disease can grow in close proximity, making it extremely challenging to distinguish them morphologically from an FTC with an invasive satellite nodule. In such cases, RAS Q61R IHC can be a valuable diagnostic tool (Fig. 3E and F). Similarly, the IHC can also highlight the multinodular infiltrative growth pattern characteristic of widely invasive FTCs with mixed macro-microfollicular architectural patterns, which can be mistaken for adenomatoid nodules within follicular nodular disease (Fig. 4A and B). Furthermore, RAS Q61R IHC can confirm the neoplastic nature of metastatic or recurrent follicular tumors with bland cytomorphological features (Fig. 4C and D). In rare cases, it can aid in identifying collision tumors composed of *RAS*-mutated follicular tumors and other *RAS*-negative tumors (Fig. 4E and F) [13].

In sporadic medullary thyroid carcinomas (MTC), in which the majority harbor *RET* mutations, up to 20% can have mutually exclusive alterations in the *RAS* family of genes, with the most common being an *HRAS* p.Q61R mutation [36]. In a recent tri-institutional cohort study of MTCs, RAS Q61R IHC was demonstrated to have exceptional sensitivity (100%), specificity (100%), positive predictive value (100%), and negative predictive value (100%) in detecting the *RAS* p.Q61R mutation in both cytologic and histologic samples of sporadic MTCs when staining was carefully ascertained to be at least weak (staining score of > 1) and/or with a membranous accentuation [37]. Membranous staining with the RAS Q61R IHC was found to be 100% predictive of *RET*-negative germline testing, highlighting its potential role as an inexpensive and rapid modality to screen patients who will undergo *RET* germline testing for MTC.

### Pan-TRK (Clone EPR 17341)

**Fig. 5:**
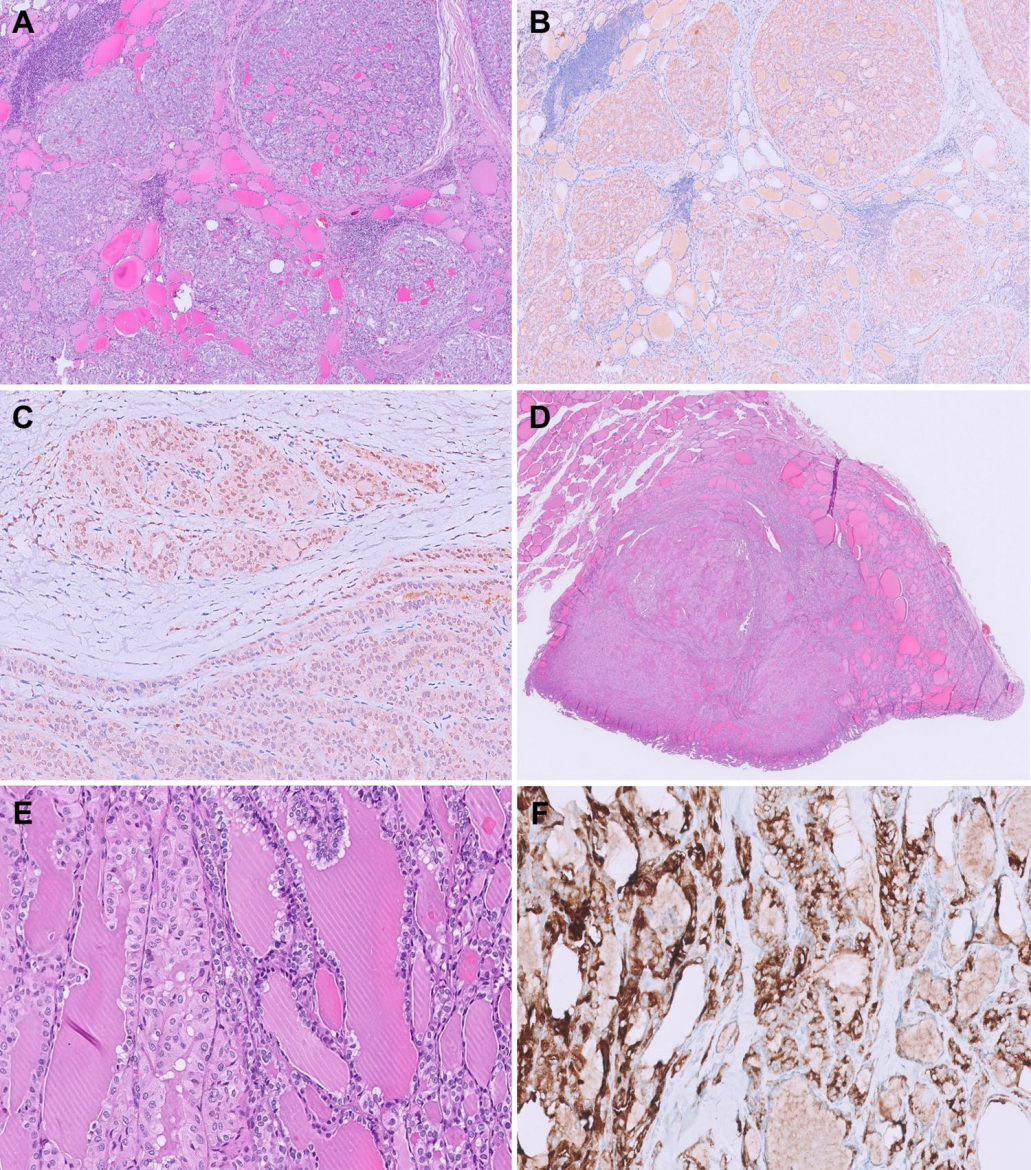
Utility of pan-TRK and ALK IHC in diagnosing thyroid carcinomas. **A** *NTRK*-rearranged PTC shows a multinodular permeative growth (H&E, 50 ×). **B** Pan-TRK IHC highlights the tumor’s multinodular pattern of invasion (50 ×). **C** Nuclear and cytoplasmic expression of pan-TRK IHC consistent with *ETV6::NTRK3* fusion (200 ×). **D** Low power view of a well-demarcated *ALK*-rearranged PTC with architectural heterogeneity (H&E, 10 ×). **E** High power view demonstrates the *ALK*-rearranged PTC with the left area showing classic PTC nuclear features juxtaposed to the right area resembling follicular nodular disease with bland cytologic features (H&E, 200 ×). **F** ALK IHC shows strong diffuse expression in the tumor (200 ×)

*NTRK* gene rearrangements are found in 2.3–3.4% of PTC, with the most common gene fusion being *ETV6::NTRK3* [1, 38]. Our previous studies found the specificity and sensitivity of pan-TRK IHC (clone EPR 17341) to be 100% and 41.7% for detecting *NTRK 1/3* rearrangements in *BRAF V600E*-negative PTCs in tissue microarray, respectively [39]. Based on a pooled analysis, pan-TRK IHC has shown lower sensitivity in detecting *NTRK3* rearrangements (65.8%) than *NTRK1* rearrangements (87.5%) [40]. Pan-TRK IHC has varied staining patterns in *NTRK*-rearranged PTCs. *ETV6::NTRK3* fusion-associated PTCs usually showed focal and limited staining in the cytoplasm and nucleus with variable staining intensity, while non-*ETV6::NTRK3* cases frequently showed a more homogeneous and diffuse staining pattern, usually in the cytoplasm with or without membranous attenuation [39]. Interestingly, the heterogeneous staining pattern in *ETV6::NTRK3* fusion-associated PTCs tends to be stronger in the tumor periphery and becomes weaker to subdiagnostic towards the center, a phenomenon that might be dismissed as artifactual in nature and considered to be non-diagnostic [41]. A knowledge of this heterogeneous staining pattern should likewise alert pathologists to the possibility of a false-negative result when interpreting pan-TRK IHC in limited biopsy samples. Similar to other kinase fusion-associated tumors, *NTRK*-rearranged PTCs tend to display non-classical histology enriched with microfollicles, multinodular permeative growth, dense intratumoral sclerosis, and subtle nuclear features, which may pose a diagnostic challenge [39, 42]. Thus, staining with pan-TRK IHC is a low-cost method to identify these cases that would require further molecular confirmation (Fig. 5A to C).

### ALK (Clones 5A4 or D5F3)

*ALK* gene rearrangements are found in 1.2% of PTCs, and the most frequent are *STRN::ALK* (45.8%) and *EML4::ALK* (31.4%). The antibody clone 5A4 has shown excellent sensitivity (100%) and modest specificity (75%) in detecting the *ALK* gene rearrangement in PTCs, seen as diffuse expression in the cytoplasm of tumor cells [43]. Additionally, we have used the D5F3 clone to identify eight cases of *ALK*-rearranged PTCs in a recent study, and six of them were confirmed through targeted RNA next-generation sequencing (NGS) (specificity 100%) [44]. *ALK* gene fusion-associated thyroid carcinomas can show subtle cytologic nuclear features resembling *RAS*-like tumors and present with a myriad of architectural patterns such as classic, classic with a predominant microfollicular growth, infiltrative follicular, and solid-trabecular [43, 44]. Rarely, a tumor can be unusually well-demarcated and have cytologic heterogeneity, with cells displaying robust PTC nuclear changes mixed with those displaying only subtle or *RAS*-like nuclear features (Fig. 5D to F). Thus, the identification of *ALK*-rearranged PTC can be diagnostically challenging without the aid of the ALK IHC.

### Applications of Next-Generation IHC in ATC

**Fig. 6:**
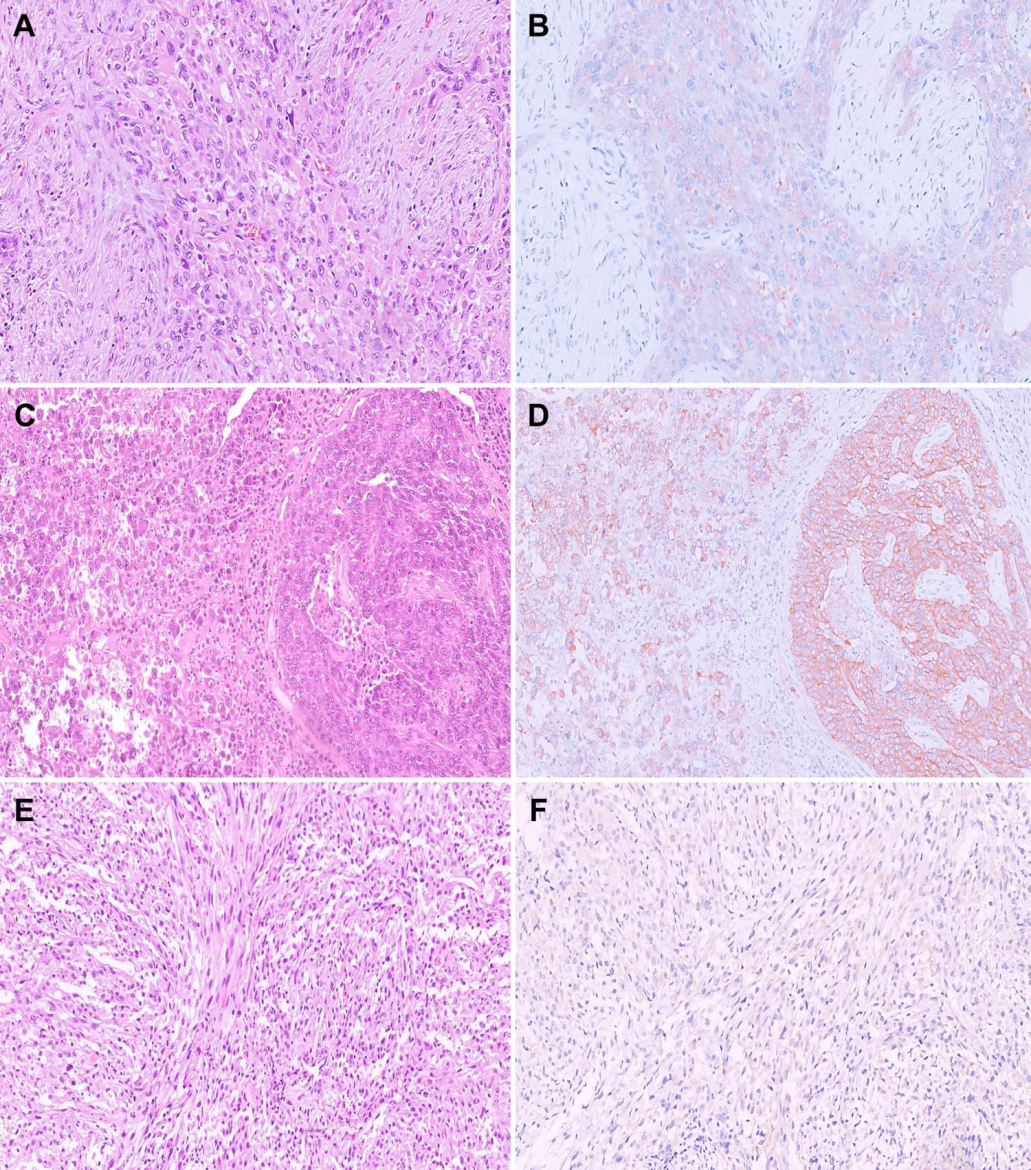
Application of next-generation IHC in the diagnosis of ATC. **A** A *BRAF* p.V600E-mutated ATC with squamous cell differentiation (H&E, 200 ×). **B** The tumor shows diffuse moderate staining with the BRAF V600E IHC (200 ×). **C** A *RAS* p.Q61R-mutated thyroid carcinoma with ATC component on the left and poorly differentiated carcinoma on the right (H&E, 200 ×). **D** RAS Q61R IHC shows diffuse staining with attenuated intensity in the ATC component (200 ×). **E** A sarcomatous ATC (H&E, 200 ×). **F** This tumor shows a diffusely faint expression of BRAF VE1 IHC and is molecularly confirmed to harbor *BRAF* p.V600E mutation by direct sequencing (200 ×)

ATC is the most lethal form of thyroid cancer, with a worldwide incidence of 1–4% and a dismal survival rate [14]. ATC may be encountered as a de novo tumor or may be accompanied by a well-differentiated or poorly differentiated thyroid carcinoma. ATC harbors *BRAF* p.V600E (25.9–45.0%) and *RAS* mutations (24.0–40.7%) as early molecular events, while *TP53* (40–80%) and *TERT* promoter mutations (30–75%) are additional late molecular events and the most frequent genetic changes seen in this tumor [45, 46]. The *BRAF* p.V600E mutation is significantly associated with ATC exhibiting a squamous cell carcinoma phenotype and/or co-existing PTC. Next-generation IHC for BRAF V600E and RAS Q61R have been employed to identify the molecular drivers of ATC, showing diffuse weak to moderate expression in the tumor cytoplasm (Fig. 6A to B). When ATC co-exists with a well-differentiated thyroid carcinoma, the staining is typically preserved in both components, consistent with tumor high-grade transformation (Fig. 6C to D). However, the expression may be diffusely attenuated in the ATC component, particularly in cases with predominantly sarcomatous differentiation (Fig. 6E and F). In such scenarios, careful evaluation of the diffusely weak expression pattern is essential, and molecular confirmation is recommended for equivocal cases. Given the critical therapeutic implications, current guidelines recommend that all ATC patients undergo rapid tumor mutational testing [47]. This includes rigorously validated *BRAF* p.V600E mutation-specific IHC as an initial screening tool, followed by rapid NGS to identify additional targetable biomarkers, particularly in *BRAF*-negative cases. The early detection of *BRAF* p.V600E-mutated ATC is crucial, as combination therapy with BRAF and MEK inhibitors has demonstrated significant clinical benefit and should be initiated without delay [14, 48]. Similarly, other actionable molecular drivers have emerged. Reports have shown a case of *ALK*-rearranged ATC with a remarkable response to crizotinib, while *NTRK*-rearranged ATCs treated with larotrectinib achieved an overall response rate of 29% [49, 50]. These findings underscore the therapeutic relevance of detecting such alterations through next-generation IHC and NGS in selected cases to guide targeted therapeutic interventions.

## Next-Generation IHC with Applications in Diagnosis and Hereditary Syndromes

### PTEN

**Fig. 7:**
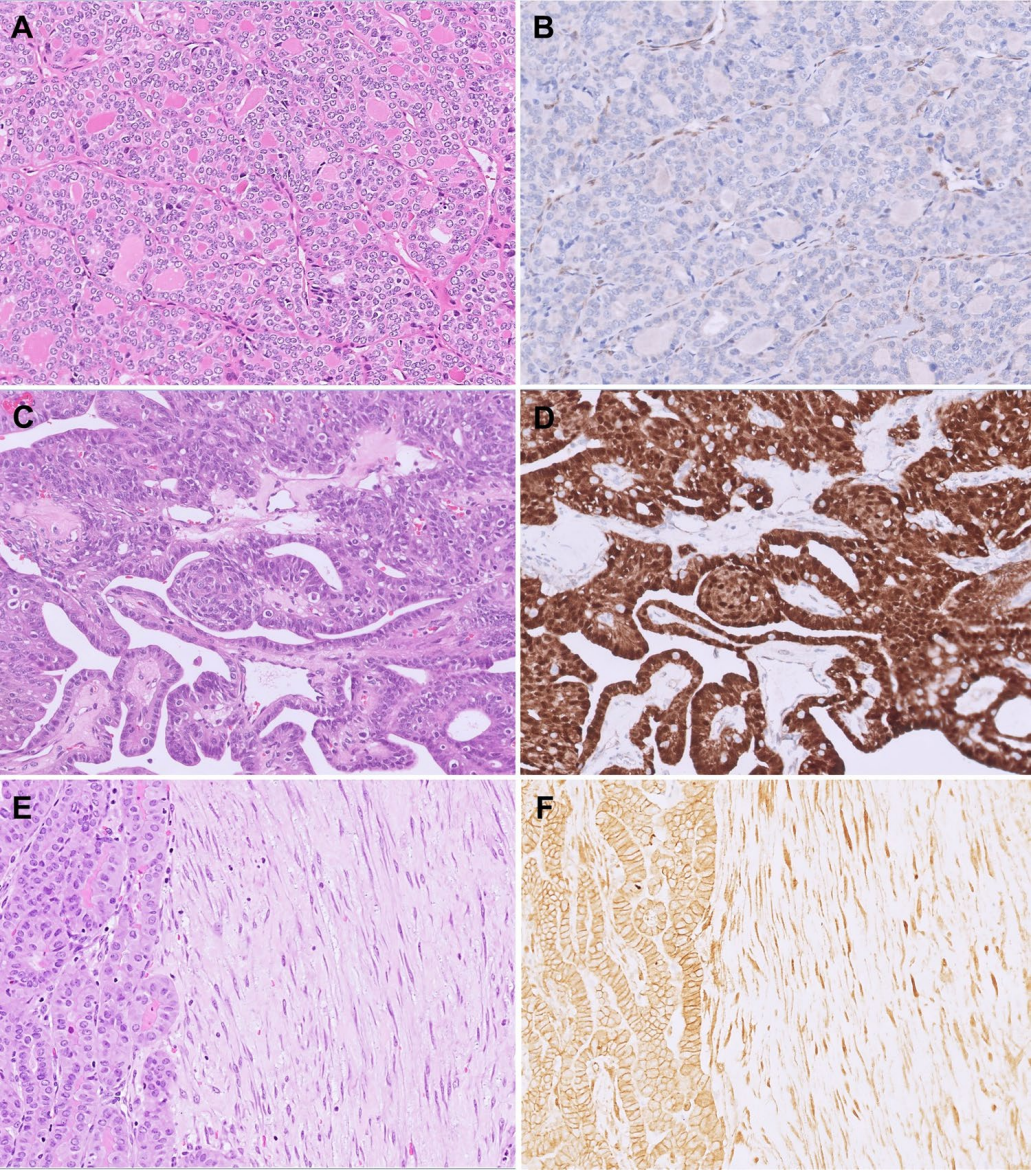
Application of next-generation IHC in diagnosing thyroid tumors associated with hereditary syndromes. **A** A minimally invasive FTC with inactivating *PTEN* mutation (H&E, 200 ×). **B** Loss of PTEN IHC expression is observed in the tumor cells, with retained expression in endothelial cells as internal positive control (200 ×). **C** CMTC displays papillary, follicular, and cribriform patterns with colloid-depleted lumina and characteristic squamous morules (H&E, 200 ×). **D** β-catenin IHC shows diffuse strong nuclear and cytoplasmic staining in tumor cells, confirming the diagnosis (200 ×). **E** PTC with fibromatosis/fasciitis-like/desmoid-type stroma (H&E, 200 ×). **F** β-catenin IHC shows diffuse strong nuclear and cytoplasmic staining in the stromal cells, confirming the diagnosis (200 ×)

PTEN hamartoma tumor syndrome (PHTS) is a rare autosomal dominant syndrome with an estimated prevalence of ~ 1:200,000, caused by germline inactivating mutations or deletions in the *PTEN* tumor suppressor gene (10q23.31) or in other functionally related genes (e.g., *PIK3CA*, *AKT1*) [14]. This results in upregulation of the PI3K/AKT/mTOR signaling pathway, promoting cell proliferation, migration, cell survival, angiogenesis, and reduced apoptosis [51, 52]. PHTS encompasses a spectrum of syndromes, including Cowden syndrome, Bannayan-Riley-Ruvalcaba syndrome, and Proteus syndrome, all characterized by multiple tissue hamartomas and an increased lifetime risk of developing benign and malignant tumors, particularly in the breast, endometrium, and thyroid. In the thyroid, PHTS manifests as multiple, bilateral cellular nodules with a wide range of benign and malignant morphologies and may be accompanied by fatty infiltration, C-cell hyperplasia, and lymphocytic thyroiditis as additional distinguishing features [4, 51]. Notably, the adenomas in PTHS can progress to carcinomas. The lifetime risk of thyroid carcinoma in PTHS ranges from 14 to 38% [51]. Thus, identifying affected families is essential for surveillance and potential prophylactic thyroidectomy [4, 14, 51]. In thyroid nodules associated with PTHS, the germline mutation is accompanied by a second-hit somatic alteration in the wild-type *PTEN* allele, resulting in the loss of PTEN protein expression, which can be detected by IHC [4, 51–53]. Loss of PTEN IHC expression in all or a subset of nodules has shown to be sensitive (100%) and specific (92.3%) for PHTS but requires further clinical work-up and confirmation [51]. Additionally, *PTEN*-inactivating mutations are found to be the driver alterations in 5–10% of sporadic FTCs and a subset of high-grade thyroid carcinomas [14]. Conveniently, in nodules with loss of PTEN IHC expression, the background normal follicular cells and endothelial cells retain the expression, acting as internal positive controls (Fig. 7A and B).

### β-Catenin

Familial adenomatous polyposis (FAP) is a rare autosomal dominant syndrome with a prevalence of 1:8000–10,000 that is caused by germline inactivating mutations in the *APC* tumor suppressor gene (5q22.2) [14]. This predisposes to numerous colorectal adenomas, an almost invariable risk of developing colorectal adenocarcinoma, and an increased risk of developing other tumors, including cribriform morular thyroid carcinoma (CMTC) [4, 14, 51]. Although CMTCs can occur sporadically, 39–53% of cases are associated with FAP [14, 54]. In up to 30–40% of affected individuals, CMTC is diagnosed before the recognition of FAP, underscoring the importance of its accurate diagnosis [51, 54]. Previously classified as a variant of PTC, CMTC is now recognized as a distinct entity due to its unique morphologic, immunophenotypic, and molecular features [55, 56]. CMTC predominantly affects young women and is often well-defined and encapsulated. Histologically, it shows a blending of papillary, follicular, cribriform, trabecular, and solid growth patterns, along with the characteristic squamous morules. Notably, CMTC does not form colloid, and the nuclear features of PTC are only variably present (Fig. 7C). While sporadic tumors are typically solitary, CMTCs occurring in the setting of FAP are often small, multiple, and bilateral [14, 51, 55, 56]. The tumor expresses TTF-1 and peculiarly estrogen and progesterone receptors but is negative for thyroglobulin and either negative or weakly positive for PAX8, suggesting a lack of definitive follicular cell differentiation. Molecularly, CMTC is driven by genetic alterations in the Wnt/β-catenin pathway. In FAP-associated multicentric tumors, a second-hit somatic mutation in the *APC* gene is commonly observed, though alterations in *KMT2C* and *KMT2D* have also been reported [14, 51]. In sporadic cases, somatic mutations in functionally equivalent genes, including *APC* (exon 15), *CTNNB1* (exon 3), and *AXIN1* (exons 1 and 7), are reported [14, 51]. A defining molecular hallmark of CMTC is an abnormal accumulation of β-catenin, with cytoplasmic and subsequent nuclear translocation, detected by the IHC as strong diffuse nuclear and cytoplasmic staining in tumor cells (Fig. 7D) [4, 51, 54, 56]. This contrasts with the membranous staining pattern observed in normal follicular cells and other thyroid tumors. β-Catenin IHC is therefore essential for diagnosing both sporadic and familial CMTC and may further facilitate the identification of patients with FAP. These defining clinicopathologic and molecular features have led to the reclassification of CMTC as a distinct entity separate from PTC and positioned within the group “thyroid tumors of uncertain histogenesis” in the latest WHO classification [14]. Aside from its association with FAP, the distinction of CMTC from PTC is clinically relevant as the lack of unequivocal follicular cell differentiation questions the potential benefits of serum thyroglobulin measurements for tumor surveillance and RAI-related adjuvant therapies [55, 56].

Lastly, β-catenin facilitates the diagnosis of the rare PTC with fibromatosis/fasciitis-like/desmoid-type stroma [14]. This biphasic tumor subtype consists of a conventional PTC intimately admixed with a cytologically bland fibroblastic-myofibroblastic proliferation that resembles a scar, nodular fasciitis, or desmoid-type fibromatosis (Fig. 7E). The spindle cells express SMA and are negative for PAX8, TTF-1, thyroglobulin, and pancytokeratin. As as defining feature, the spindle cells display strong nuclear and cytoplasmic expression of β-catenin IHC (Fig. 7F), which corresponds to underlying *CTNNB1* mutations found in the majority of cases [57, 58]. The aforementioned features help distinguish this tumor from the spindle cell and paucicellular anaplastic thyroid carcinoma arising in a PTC [14]. Though treated similarly to conventional PTC, the accurate diagnosis may have clinical implications as the desmoid fibromatosis stroma will likely not respond to iodine therapy, and its presence may necessitate wider margins to prevent recurrence [57]. Furthermore, the metastasis has shown to invariably consist of the stromal component, which may cause further diagnostic issues on recurrence [59].

## Conclusion

**Fig. 8:**
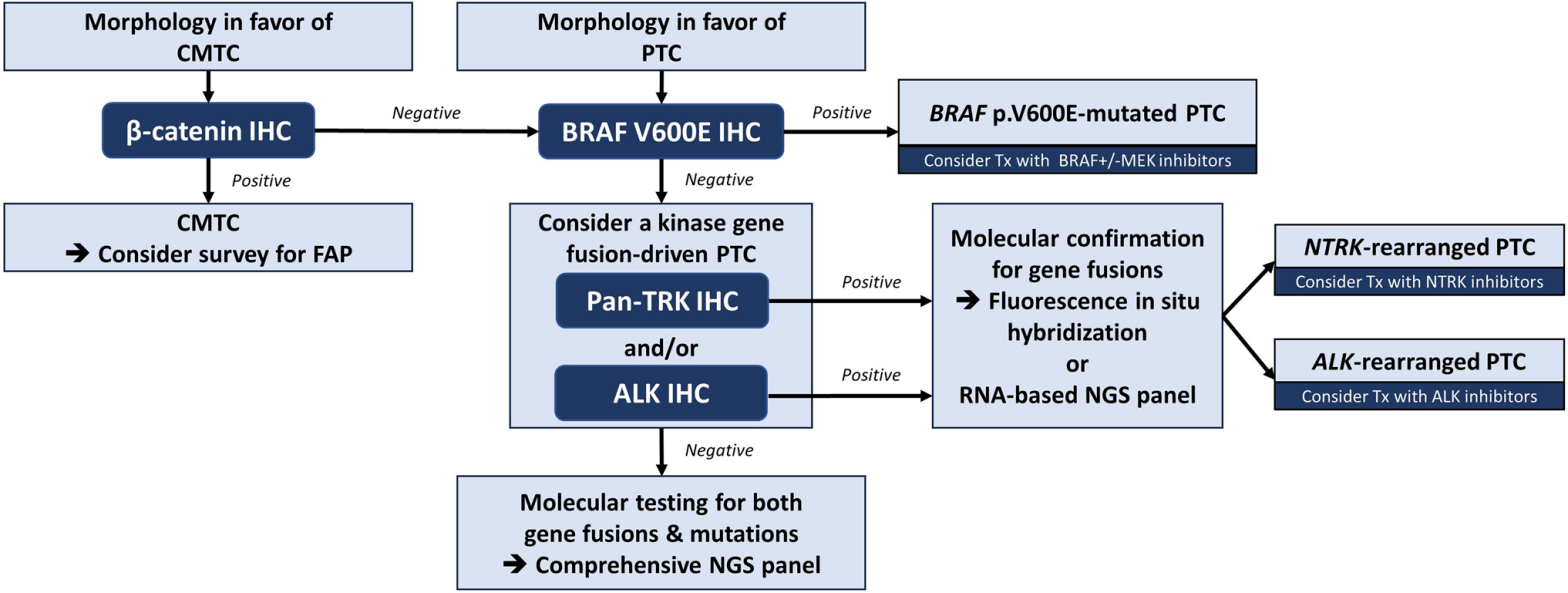
Proposed diagnostic algorithm for the molecular classification of PTC and diagnosis of CMTC

**Fig. 9:**
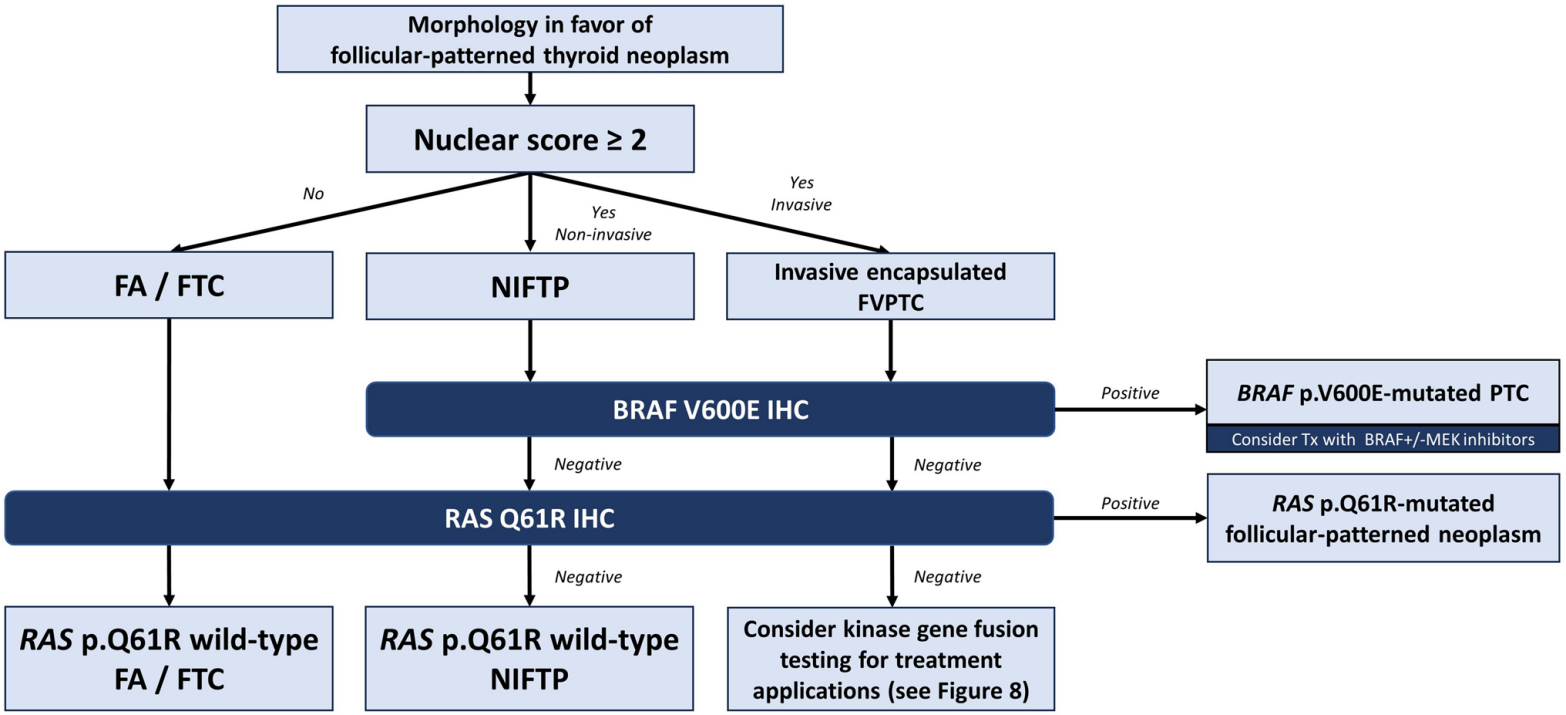
Proposed diagnostic algorithm for the molecular classification of follicular-patterned thyroid neoplasms

The next-generation IHC for BRAF V600E, RAS Q61R, pan-TRK, ALK, PTEN, and β-catenin has promising roles in the diagnosis and molecular classification of thyroid carcinomas. In well-differentiated thyroid carcinomas, these stains are invaluable for highlighting tumor extent, invasion, and lymph node involvement. They also help differentiate benign intranodal thyroid inclusions from metastatic tumor deposits, identify multicentric thyroid carcinomas with discordant molecular drivers or collision tumors, and screen patients for potential predisposing hereditary syndromes. A summary of these diagnostic applications is provided in Table 1, and proposed diagnostic algorithms for the molecular classification of PTC and follicular-patterned neoplasms are provided in Figs. 8 and 9, respectively. In ATC, these stains allow fast and accurate identification of the underlying molecular drivers, which has revolutionized the treatment of this aggressive malignancy. Collectively, these advances enhance diagnostic accuracy, facilitate precise pathologic staging and risk stratification, support the appropriate selection of targeted therapies for advanced or radioiodine-refractory disease, and improve the screening and management of patients with hereditary syndromes. Finally, these tools offer novel solutions to longstanding controversies and diagnostic challenges in thyroid pathology, such as intranodal benign thyroid inclusions and collision tumors.

**Table 1:** Summary of the diagnostic applications of next-generation IHC in well-differentiated thyroid tumors

IHC	Application
BRAF V600E (clone VE1)	Highlight tumor extent and unique patterns of invasion
	Identify multicentric PTCs with discordant molecular drivers
	Identify rare collision tumors consisting of molecularly discordant PTC
	Highlight isolated tumor clusters in lymph nodes
	Distinguish benign intranodal thyroid inclusions from metastatic PTC
	Exclusion of a NIFTP
RAS Q61R (clone SP174)	Assist in the accurate demonstration of tumor border, infiltration, capsular and/or vascular invasion, and multifocality
	Help confirm the diagnosis of minimally invasive FTC and distinguish it from *RAS*-mutated hyperplastic nodules
	Help diagnose the multinodular permeative growth of widely invasive FTC that may deceptively mimic adenomatoid nodules of follicular nodular disease
	Confirm the distant metastasis or recurrence of a *RAS*-like thyroid carcinoma with bland cytoarchitectural features
	Identify rare collisions of *RAS*-mutated follicular tumors
	Diagnose sporadic MTCs with *RAS* p.Q61R mutation to triage patients who will undergo *RET* germline mutation testing
Pan-TRK (clone EPR 17341)	Help diagnose *NTRK*-rearranged PTCs as a surrogate for the presence of gene fusion and highlight the tumor area
ALK (clones 5A4 and D5F3)	Help diagnose *ALK*-rearranged PTCs as a surrogate for the presence of gene fusion and highlight the tumor area
PTEN	Help diagnose *PTEN*-mutated follicular nodules and screen patients for potential PTHS
β-catenin	Confirm the diagnosis of CMTC and screen patients for potential FAP syndrome
	Confirm the diagnosis of PTC with fibromatosis/fasciitis-like/desmoid-type stroma

## Data Availability

No datasets were generated or analysed during the current study.

## References

[1] The Cancer Genome Atlas Research Network (2014) Integrated genomic characterization of papillary thyroid carcinoma. Cell 159: 676-690. 25417114 10.1016/j.cell.2014.09.050 PMC4243044

[2] Shonka DC, Jr., Ho A, Chintakuntlawar AV et al. (2022) American Head and Neck Society Endocrine Surgery Section and International Thyroid Oncology Group consensus statement on mutational testing in thyroid cancer: Defining advanced thyroid cancer and its targeted treatment. Head Neck 44: 1277-1300. 35274388 10.1002/hed.27025 PMC9332138

[3] Chu YH (2023) This is Your Thyroid on Drugs: Targetable Mutations and Fusions in Thyroid Carcinoma. Surg Pathol Clin 16: 57-73. 36739167 10.1016/j.path.2022.09.007

[4] Guilmette J, Nose V (2018) Hereditary and familial thyroid tumours. Histopathology 72: 70-81. 29239041 10.1111/his.13373

[5] Hornick JL (2021) Replacing Molecular Genetic Testing With Immunohistochemistry Using Antibodies That Recognize the Protein Products of Gene Rearrangements: “Next-generation” Immunohistochemistry. Am J Surg Pathol 45: 584-586. 33399342 10.1097/PAS.0000000000001663

[6] Hang JF, Chen JY, Kuo PC et al. (2023) A Shift in Molecular Drivers of Papillary Thyroid Carcinoma Following the 2017 World Health Organization Classification: Characterization of 554 Consecutive Tumors With Emphasis on BRAF-Negative Cases. Mod Pathol 36: 100242. 37307878 10.1016/j.modpat.2023.100242

[7] Capper D, Preusser M, Habel A et al. (2011) Assessment of BRAF V600E mutation status by immunohistochemistry with a mutation-specific monoclonal antibody. Acta Neuropathol 122: 11-19. 21638088 10.1007/s00401-011-0841-z

[8] Ritterhouse LL, Barletta JA (2015) BRAF V600E mutation-specific antibody: A review. Semin Diagn Pathol 32: 400-408. 25744437 10.1053/j.semdp.2015.02.010

[9] Hang JF, Li AF, Chang SC, Liang WY (2016) Immunohistochemical detection of the BRAF V600E mutant protein in colorectal cancers in Taiwan is highly concordant with the molecular test. Histopathology 69: 54-62. 26588428 10.1111/his.12903

[10] Parker KG, White MG, Cipriani NA (2020) Comparison of Molecular Methods and BRAF Immunohistochemistry (VE1 Clone) for the Detection of BRAF V600E Mutation in Papillary Thyroid Carcinoma: A Meta-Analysis. Head Neck Pathol 14: 1067-1079. 32358715 10.1007/s12105-020-01166-8 PMC7669962

[11] Jung CK, Kang YG, Bae JS, Lim DJ, Choi YJ, Lee KY (2010) Unique patterns of tumor growth related with the risk of lymph node metastasis in papillary thyroid carcinoma. Mod Pathol 23: 1201-1208. 20543822 10.1038/modpathol.2010.116

[12] Tallini G, de Biase D, Durante C et al. (2015) BRAF V600E and risk stratification of thyroid microcarcinoma: a multicenter pathological and clinical study. Mod Pathol 28: 1343-1359. 26271724 10.1038/modpathol.2015.92

[13] Rivera JP, Yeh YC, Chen PC, Hang JF (2024) Multifocal Papillary Thyroid Carcinomas With Discordant Molecular Drivers: Emphasizing the Morphology and Collision Tumors. Am J Surg Pathol 48: 1359-1371. 38818543 10.1097/PAS.0000000000002256

[14] WHO Classification of Tumours Editorial Board. Endocrine and neuroendocrine tumours. 5th ed. Lyon (France): International Agency for Research on Cancer 2022

[15] Zhu Z, Ciampi R, Nikiforova MN, Gandhi M, Nikiforov YE (2006) Prevalence of RET/PTC rearrangements in thyroid papillary carcinomas: effects of the detection methods and genetic heterogeneity. J Clin Endocrinol Metab 91: 3603-3610. 16772343 10.1210/jc.2006-1006

[16] Henderson YC, Shellenberger TD, Williams MD et al. (2009) High rate of BRAF and RET/PTC dual mutations associated with recurrent papillary thyroid carcinoma. Clin Cancer Res 15: 485-491. 19147753 10.1158/1078-0432.CCR-08-0933 PMC3038608

[17] Guerra A, Sapio MR, Marotta V et al. (2012) The primary occurrence of BRAF(V600E) is a rare clonal event in papillary thyroid carcinoma. J Clin Endocrinol Metab 97: 517-524. 22170714 10.1210/jc.2011-0618

[18] Zou M, Baitei EY, Alzahrani AS et al. (2014) Concomitant RAS, RET/PTC, or BRAF mutations in advanced stage of papillary thyroid carcinoma. Thyroid 24: 1256-1266. 24798740 10.1089/thy.2013.0610 PMC4106383

[19] Finkel A, Liba L, Simon E et al. (2016) Subclonality for BRAF Mutation in Papillary Thyroid Carcinoma Is Associated With Earlier Disease Stage. J Clin Endocrinol Metab 101: 1407-1413. 26835544 10.1210/jc.2015-4031

[20] Ghossein RA, Katabi N, Fagin JA (2013) Immunohistochemical detection of mutated BRAF V600E supports the clonal origin of BRAF-induced thyroid cancers along the spectrum of disease progression. J Clin Endocrinol Metab 98: E1414-1421. 23775351 10.1210/jc.2013-1408 PMC6287446

[21] Leeman-Neill RJ, Kelly LM, Liu P et al. (2014) ETV6-NTRK3 is a common chromosomal rearrangement in radiation-associated thyroid cancer. Cancer 120: 799-807. 24327398 10.1002/cncr.28484 PMC3947712

[22] Xing M, Haugen BR, Schlumberger M (2013) Progress in molecular-based management of differentiated thyroid cancer. Lancet 381: 1058-1069. 23668556 10.1016/S0140-6736(13)60109-9 PMC3931461

[23] Meyer JS, Steinberg LS (1969) Microscopically benign thyroid follicles in cervical lymph nodes. Serial section study of lymph node inclusions and entire thyroid gland in 5 cases. Cancer 24: 302-311. 5796776 10.1002/1097-0142(196908)24:2<302::aid-cncr2820240213>3.0.co;2-v

[24] Triantafyllou A, Williams MD, Angelos P et al. (2016) Incidental findings of thyroid tissue in cervical lymph nodes: old controversy not yet resolved? Eur Arch Otorhinolaryngol 273: 2867-2875. 26459007 10.1007/s00405-015-3786-3 PMC5525538

[25] Chuang YC, Kuo YJ, Hang JF (2024) Intranodal thyroid inclusions revisited: a morphological analysis and application of BRAF VE1 immunohistochemistry. Histopathology [online ahead of print] 10.1111/his.15394 PMC11964582 39687984

[26] Hanrahan AJ, Chen Z, Rosen N, Solit DB (2024) BRAF - a tumour-agnostic drug target with lineage-specific dependencies. Nat Rev Clin Oncol 21: 224-247. 38278874 10.1038/s41571-023-00852-0 PMC11857949

[27] Xing M (2013) Molecular pathogenesis and mechanisms of thyroid cancer. Nat Rev Cancer 13: 184-199. 23429735 10.1038/nrc3431 PMC3791171

[28] Nikiforov YE, Seethala RR, Tallini G et al. (2016) Nomenclature Revision for Encapsulated Follicular Variant of Papillary Thyroid Carcinoma: A Paradigm Shift to Reduce Overtreatment of Indolent Tumors. JAMA Oncol 2: 1023-1029. 27078145 10.1001/jamaoncol.2016.0386 PMC5539411

[29] Hernandez-Prera JC, Wenig BM (2024) RAS-Mutant Follicular Thyroid Tumors: A Continuous Challenge for Pathologists. Endocr Pathol 35: 167-184. 38888731 10.1007/s12022-024-09812-5

[30] Landa I, Cabanillas ME (2024) Genomic alterations in thyroid cancer: biological and clinical insights. Nat Rev Endocrinol 20: 93-110. 38049644 10.1038/s41574-023-00920-6

[31] Guan H, Toraldo G, Cerda S et al. (2020) Utilities of RAS Mutations in Preoperative Fine Needle Biopsies for Decision Making for Thyroid Nodule Management: Results from a Single-Center Prospective Cohort. Thyroid 30: 536-547. 31996097 10.1089/thy.2019.0116

[32] Ohori NP, Nishino M (2023) Follicular Neoplasm of Thyroid Revisited: Current Differential Diagnosis and the Impact of Molecular Testing. Adv Anat Pathol 30: 11-23. 36102526 10.1097/PAP.0000000000000368

[33] Saliba M, Katabi N, Dogan S, Xu B, Ghossein RA (2021) NRAS Q61R immunohistochemical staining in thyroid pathology: sensitivity, specificity and utility. Histopathology 79: 650-660. 33960437 10.1111/his.14396 PMC8458224

[34] Alzumaili BA, Fisch AS, Faquin WC, Nose V, Randolph GW, Sadow PM (2024) Detection of RAS p.Q61R by Immunohistochemistry in Practice: A Clinicopathologic Study of 217 Thyroid Nodules with Molecular Correlates. Endocr Pathol 35: 219-229. 39096324 10.1007/s12022-024-09821-4

[35] Crescenzi A, Fulciniti F, Bongiovanni M, Giovanella L, Trimboli P (2017) Detecting N-RAS Q61R Mutated Thyroid Neoplasias by Immunohistochemistry. Endocr Pathol 28: 71-74. 28064410 10.1007/s12022-016-9466-z

[36] Moura MM, Cavaco BM, Pinto AE, Leite V (2011) High prevalence of RAS mutations in RET-negative sporadic medullary thyroid carcinomas. J Clin Endocrinol Metab 96: E863-868. 21325462 10.1210/jc.2010-1921

[37] Deyette B, Lubin DJ, Cheriyan AM et al. (2024) The Potential Utility of RAS Q61R Immunohistochemistry as a Screening Tool in Pre-operative Fine Needle Aspirates of Medullary Thyroid Carcinoma. Endocr Pathol 35: 385-396. 39630334 10.1007/s12022-024-09839-8

[38] Liang J, Cai W, Feng D et al. (2018) Genetic landscape of papillary thyroid carcinoma in the Chinese population. J Pathol 244: 215-226. 29144541 10.1002/path.5005

[39] Lee YC, Chen JY, Huang CJ, Chen HS, Yang AH, Hang JF (2020) Detection of NTRK1/3 Rearrangements in Papillary Thyroid Carcinoma Using Immunohistochemistry, Fluorescent In Situ Hybridization, and Next-Generation Sequencing. Endocr Pathol 31: 348-358. 32880785 10.1007/s12022-020-09648-9

[40] Hang JF, Lee YC (2023) How Sensitive is Pan-TRK Immunohistochemistry for Detecting NTRK Fusions in Papillary Thyroid Carcinoma? Mod Pathol 36: 100222. 37336120 10.1016/j.modpat.2023.100222

[41] Conde E, Hernandez S, Sanchez E et al. (2021) Pan-TRK Immunohistochemistry: An Example-Based Practical Approach to Efficiently Identify Patients With NTRK Fusion Cancer. Arch Pathol Lab Med 145: 1031-1040. 33112951 10.5858/arpa.2020-0400-RA

[42] Chu YH, Wirth LJ, Farahani AA et al. (2020) Clinicopathologic features of kinase fusion-related thyroid carcinomas: an integrative analysis with molecular characterization. Mod Pathol 33: 2458-2472. 32737449 10.1038/s41379-020-0638-5 PMC7688509

[43] Chou A, Fraser S, Toon CW et al. (2015) A detailed clinicopathologic study of ALK-translocated papillary thyroid carcinoma. Am J Surg Pathol 39: 652-659. 25501013 10.1097/PAS.0000000000000368 PMC4415964

[44] Shih KP, Lee YC, Tsai JJ et al. (2024) Clinicopathologic Features and Cytologic Correlation of ALK-Rearranged Papillary Thyroid Carcinoma: A Series of Eight Cases. Endocr Pathol 35: 134-146. 38642308 10.1007/s12022-024-09808-1 PMC11176248

[45] Xu B, Fuchs T, Dogan S et al. (2020) Dissecting Anaplastic Thyroid Carcinoma: A Comprehensive Clinical, Histologic, Immunophenotypic, and Molecular Study of 360 Cases. Thyroid 30: 1505-1517. 32284020 10.1089/thy.2020.0086 PMC7583343

[46] Lai WA, Liu CY, Lin SY, Chen CC, Hang JF (2020) Characterization of Driver Mutations in Anaplastic Thyroid Carcinoma Identifies RAS and PIK3CA Mutations as Negative Survival Predictors. Cancers (Basel) 12: 1973. 32698386 10.3390/cancers12071973 PMC7409295

[47] Mete O, Boucher A, Schrader KA et al. (2024) Consensus Statement: Recommendations on Actionable Biomarker Testing for Thyroid Cancer Management. Endocr Pathol 35: 293-308. 39579327 10.1007/s12022-024-09836-x PMC11659332

[48] Subbiah V, Kreitman RJ, Wainberg ZA et al. (2018) Dabrafenib and Trametinib Treatment in Patients With Locally Advanced or Metastatic BRAF V600-Mutant Anaplastic Thyroid Cancer. J Clin Oncol 36: 7-13. 29072975 10.1200/JCO.2017.73.6785 PMC5791845

[49] Waguespack SG, Drilon A, Lin JJ et al. (2022) Efficacy and safety of larotrectinib in patients with TRK fusion-positive thyroid carcinoma. Eur J Endocrinol 186: 631-643. 35333737 10.1530/EJE-21-1259 PMC9066591

[50] Godbert Y, Henriques de Figueiredo B, Bonichon F et al. (2015) Remarkable Response to Crizotinib in Woman With Anaplastic Lymphoma Kinase-Rearranged Anaplastic Thyroid Carcinoma. J Clin Oncol 33: e84-87. 24687827 10.1200/JCO.2013.49.6596

[51] Cameselle-Teijeiro JM, Mete O, Asa SL, LiVolsi V (2021) Inherited Follicular Epithelial-Derived Thyroid Carcinomas: From Molecular Biology to Histological Correlates. Endocr Pathol 32: 77-101. 33495912 10.1007/s12022-020-09661-y PMC7960606

[52] Hartsough E, DeSimone MS, Lorenzo ME, Dias-Santagata D, Nose V, Hoang MP (2024) Utilizing PTEN immunohistochemistry as a screening test for Cowden syndrome. Am J Clin Pathol 161: 490-500. 38206110 10.1093/ajcp/aqad177

[53] Barletta JA, Bellizzi AM, Hornick JL (2011) Immunohistochemical staining of thyroidectomy specimens for PTEN can aid in the identification of patients with Cowden syndrome. Am J Surg Pathol 35: 1505-1511. 21921783 10.1097/PAS.0b013e31822fbc7d

[54] Andrici J, Gill AJ, Hornick JL (2018) Next generation immunohistochemistry: Emerging substitutes to genetic testing? Semin Diagn Pathol 35: 161-169. 28662997 10.1053/j.semdp.2017.05.004

[55] Cameselle-Teijeiro JM, Peteiro-Gonzalez D, Caneiro-Gomez J et al. (2018) Cribriform-morular variant of thyroid carcinoma: a neoplasm with distinctive phenotype associated with the activation of the WNT/beta-catenin pathway. Mod Pathol 31: 1168-1179. 29785019 10.1038/s41379-018-0070-2

[56] Boyraz B, Sadow PM, Asa SL, Dias-Santagata D, Nose V, Mete O (2021) Cribriform-Morular Thyroid Carcinoma Is a Distinct Thyroid Malignancy of Uncertain Cytogenesis. Endocr Pathol 32: 327-335. 34019236 10.1007/s12022-021-09683-0 PMC9353615

[57] Rebecchini C, Nobile A, Piana S et al. (2017) Papillary thyroid carcinoma with nodular fasciitis-like stroma and beta-catenin mutations should be renamed papillary thyroid carcinoma with desmoid-type fibromatosis. Mod Pathol 30: 236-245. 27713418 10.1038/modpathol.2016.173

[58] Suster D, Michal M, Nishino M et al. (2020) Papillary thyroid carcinoma with prominent myofibroblastic stromal component: clinicopathologic, immunohistochemical and next-generation sequencing study of seven cases. Mod Pathol 33: 1702-1711. 32291398 10.1038/s41379-020-0539-7

[59] Takada N, Hirokawa M, Ito M et al. (2017) Papillary thyroid carcinoma with desmoid-type fibromatosis: A clinical, pathological, and immunohistochemical study of 14 cases. Endocr J 64: 1017-1023. 28794344 10.1507/endocrj.EJ17-0242

